# The influence of PFK-II overexpression on neuroblastoma patients’ survival may be dependent on the particular isoenzyme expressed, PFKFB3 or PFKFB4

**DOI:** 10.1186/s12935-019-1005-9

**Published:** 2019-11-14

**Authors:** Sonia E. Trojan, Michał J. Markiewicz, Katarzyna Leśkiewicz, Kinga A. Kocemba-Pilarczyk

**Affiliations:** 1 Faculty of Medicine, Chair of Medical Biochemistry, Jagiellonian University-Medical College, Kraków, Poland; 2 Jagiellonian University, Office of Institutional Analysis, Kraków, Poland

**Keywords:** Phosphofructokinase II, PFKFB3, PFKFB4, Cancer progression, Overall survival, Neuroblastoma

## Abstract

**Background/Aim:**

During cancer progression metabolic reprogramming is observed in parallel to the alternation in transcriptional profiles of malignant cells. Recent studies suggest that metabolic isoenzymes of phosphofructokinase II (PFK-II) – PFKFB3 and PFKFB4, often induced in hypoxic environment, significantly contribute to enhancement of glucose metabolism and in consequence cancer progression.

**Materials and methods:**

Using the publicly available data deposited in the R2 data base we performed a Kaplan–Meyer analysis for cancer patients divided into groups with high and low expression levels of PFKFB3/4, determined based on the median.

**Results:**

Our data showed that high PFKFB3/4 expression significantly correlates with shorter overall survival in several cancers. Moreover, we found that neuroblastoma patients with poor overall survival and evidence free survival are characterized by high PFKFB3 and at the same time low PFKFB4 expression, whereas patients with high PFKFB4 expressions are characterized by significantly better overall survival/evidence free survival rates.

**Conclusion:**

Our analysis clearly indicates that expression of PFKFB3/4 isoenzymes may have a key prognostic value for several cancers. What’s more, it seems that in neuroblastoma the prognostic value of PFK-II may be dependent on the relation between PFKFB3 and PFKFB4 isoenzyme expression, indicating that further studies analyzing the role of both cancer specific PFK-II isoenzymes are highly desired.

## Background

Nowadays, the metabolic reprogramming of cancer cells is considered a very promising field for development of novel therapeutic strategies. Apparently, the most evident alterations in cancer metabolism are related to glucose breakdown. As initially observed by Otto Warburg, in comparison to normal cells, the cancer cells ATP is generated mainly by glycolysis, even in normoxic conditions [1, 2]. Although glycolysis is less efficient than oxidative phosphorylation, it allows the generation of ATP at a much faster pace, which is highly beneficial for proliferating cancer cells. Additionally, elevation of glycolysis enables sufficient production levels of glycolytic intermediates required for de novo nucleotide, lipid, amino acid, and NADPH synthesis. The role of enhanced glucose breakdown in carcinogenesis is well confirmed by studies, showing that attenuation of glycolysis inhibits growth and decreases survival of cancer cells [3, 4]. Without doubt, an attractive anti-cancer approach would be to target glycolysis by inhibiting the aforementioned enzymes crucial for this process. Nevertheless, to do so, there must be a significant difference in the particular enzyme expression and/or activity between cancer cells and normal proliferating cells. In fact, studies in recent years revealed that PFKFB3/PFKFB4 isoenzymes are excellent candidates for glycolysis targeting, especially in cancer cells [5, 6]. PFKFB3 and PFKFB4 are cancer specific isoenzymes of the bifunctional 6-phosphofructo-2-kinase/fructose-2,6-bisphosphatase (PFKFB) enzyme group, responsible for controlling the steady-state cytoplasmic levels of fructose-2,6-bisphosphate, which allosterically activates phosphofructokinase-I (PFK-I), the key enzyme that catalyzes the rate-limiting step of glycolysis [5, 7]. Several in vitro studies revealed that targeting PFKFB3 and PFKFB4 in cancer cells results in glycolysis inhibition and in consequence, attenuation of tumor growth [8–12]. Without doubt, it is worth noting that PFKFB4 and PFKFB3 isoenzymes are not only involved in glucose metabolism but may also regulate other processes crucial for carcinogenesis, having a multi-level anti-cancer effect [13–16]. Taking into consideration all of the aforementioned, and the fact that there is limited clinical data related to this topic, in our current paper, using publicly available data sets deposited in the R2 data base, we analyzed the influence of PFKFB3 and PFKFB4 expression on overall survival of 49 independent, available cancer data sets. The results of the analysis indicates the importance of PFKFB3/4 co-expression for the clinical outcome of cancer patients.

## Materials and methods

### R2 database

The R2 database (http://r2.amc.nl) is a simple to use web-based genomics analysis and visualization application developed at the Department of Oncogenomics in the Academic Medical Center (AMC) in Amsterdam, Netherlands [17]. It allows researchers to perform a range of analyses based on well annotated datasets. For the purpose of this study we have chosen the Kaplan–Meier analysis of PFKFB3/PFKFB4 gene expression in the panel of cancer data sets. The gene expression cutoff value was chosen as a median over the entire dataset to ensure all analyses were based on the same cutoff.

### Data validation and statistical analysis

For validation purposes the gene expression and survival data of 498 neuroblastoma patients (GSE62564) were downloaded from the NCBI Gene Expression Omnibus (GEO), a public repository of microarray data [18]. Based on the median expression level of PFKFB4 and PFKFB3 genes, the patients were divided into four groups, namely: low PFKFB3/low PFKFB4 (G1), low PFKFB3/high PFKFB4 (G2), high PFKFB3/low PFKFB4 (G3) and high PFKFB3/high PFKFB4 (G4) respectively, the survival curves were plotted using the Kaplan–Meier method and compared between each other using the log-rank test with Bonferroni correction (p value below 0.05/6 was consider significant). Kaplan–Meyer analysis and univariate Cox proportional hazards regression were performed using GraphPad Prism 5.01 (GraphPad Software, La Jolla, CA, USA).

## Results

### PFKFB3 and PFKFB4 expression-based survival analysis in a panel of tumors using the R2 data base

Initially, using the R2 data base we analyzed the relation between PFKFB3 and PFKFB4 expression and the overall survival rate in the panel of cancers. The only criterium for this analysis was availability of overall survival and expression data. The analyzed panel composed of 49 data sets (Table 1), where some tumors were represented more than once. In every single data set, the patients were divided into two groups based on the median expression of PFKFB3 and/or PFKFB4. The difference in survival between these two groups was analyzed by a log rank test. Probability values below 0.05 were considered statistically significant and those between 0.05 and 0.1 were marked as an indication of a trend. Our analysis revealed that in the case of Glioma and Liver hepatocellular carcinoma patients with high PFKFB4 expression have significantly lower overall survival rates in comparison to patients with low expression of PFKFB4 (Table 1). As for PFKFB3, we have found that in kidney renal papillary cell carcinoma patients high expression of this isoenzyme was significantly correlated with lower overall survivability. Interestingly, in cervical squamous cell carcinoma we revealed that patients with high expression of both, PFKFB3 and PFKFB4 have significantly worse overall survival in comparison to the ones with low expression. Although the overexpressed PFKFB3/4 isoenzymes are considered oncogenic, mantle cell lymphoma, ovarian and pancreatic adenocarcinoma data sets have revealed that patients with low expression of PFKFB3 enzyme have significantly worse survival rates in comparison to the ones with high expression (Table 1).

**Table 1:** Analysis of the relation between PFKFB3 and PFKFB4 expression and the overall survival rate in the panel of cancers

Cancer type	Patient number	Source	P value for PFKFB3	P value for PFKFB4
Acute myeloid leukemia	422	GEO ID:GSE37642	0.713	0.704
Adrenocortical carcinoma	79	TCGA ID:ND	*0.083*^*a*^	*0.076*^*a*^
B-cell lymphoma	414	GEO ID:GSE10846	*0.069*^*b*^	0.528
Bladder urothelial carcinoma	224	GEO ID:GSE32894	0.508	0.331
Bladder urothelial carcinoma	408	TCGA ID:BLCA	0.555	0.352
Breast	159	GEO ID:GSE1456	0.405	0.110
Breast	130	GEO ID:GSE69031	0.527	0.913
Breast invasive carcinoma	1096	TCGA ID:BRCA	0.358	0.858
***Cervical squamous cell carcinoma***	***292***	***TCGA ID:CESC***	***0.032***^***a***^	***0.026***^***a***^
Cholangiocarcinoma	36	TCGA ID:CHOL	0.859	0.402
***Colon***	***200***	***GEO ID:GSE17538***	***0.041***^***a***^	0.775
Esophageal adenocarcinomas	70	GEO ID:GSE19417	0.854	*0.098*^*b*^
Esophageal carcinoma	183	TCGA ID:ESCA	0.814	0.384
Ewing sarcoma	85	GEO ID:GSE63157	*0.073*^*a*^	*0.094*^*a*^
Glioblastoma	80	GEO ID:GSE7696	1.000	1.000
Glioblastoma	377	ND ID:ND	0.525	0.286
Glioblastoma	504	ND ID:ND	0.964	0.387
***Glioma***	***490***	***GEO ID:GSE108474***	0.378	***1.2e−09***^***a***^
***Glioma***	***273***	***GEO ID:GSE16011***	0.129	***1.4e−10***^***a***^
***Glioma***	***50***	***GEO ID:GSE43378***	0.730	***0.028***^***a***^
Glioma pediatric	47	GEO ID:GSE19578	*0.092*^*a*^	0.625
Intrinsic glioma subtypes	95	GEO ID:GSE43107	*0.069*^*b*^	0.660
Head neck squamous cell carcinoma	520	TCGA ID:HNSC	0.640	0.875
Kidney renal clear cell carcinoma	533	TCGA ID:KIRC	0.464	0.542
***Kidney renal papillary cell carcinoma***	***290***	***TCGA ID:KIRP***	***0.012***^***a***^	*0.064*^*a*^
***Liver hepatocellular carcinoma***	***371***	***TCGA ID:LIHC***	*0.058*^*a*^	***6.6e−05***^***a***^
Lung	106	GEO ID:GSE3141	0.819	*0.100*^*a*^
Lung adenocarcinoma	515	TCGA ID:LUAD	0.814	0.961
Tumor lymphoma	162	GEO ID:GSE58445	0.315	0.486
Tumor lymphoma	470	GEO ID:GSE31312	0.431	0.555
***Mantle cell lymphoma***	***122***	***GEO ID:GSE93291***	***0.030***^***b***^	*0.070*^*a*^
Medulloblastoma	612	GEO ID:GSE85217	0.576	0.720
***Melanoma***	***214***	***GEO ID:GSE65904***	0.633	***0.048***^***a***^
Metastatic melanoma	44	GEO ID:GSE19234	0.558	0.394
Myeloma	542	GEO ID:GSE2658	*0.078*^*b*^	0.514
***Neuroblastoma***	***476***	***GEO ID:GSE45547***	***1.8e−04***^***a***^	***6.2e−04***^***b***^
***Neuroblastoma***	***498***	***GEO ID:GSE62564***	***6.8e−04***^***a***^	***1.6e−06***^***b***^
Neuroblastoma stage IV	27	GEO ID:GSE79910	0.939	0.325
***Neuroblastoma primary***	***283***	***GEO ID:GSE85047***	*0.090*^*a*^	***3.3e−03***^***b***^
***Neuroblastoma***	***247***	***ND ID:ND***	0.272	***6.1e−03***^***b***^
Neuroblastoma	88	GEO ID:GSE16476	0.398	*0.078*^*b*^
***Ovarian***	***75***	***GEO ID:GSE63885***	***3.9e−03***^***b***^	0.941
Ovarian adenocarcinoma	107	GEO ID:GSE26193	0.371	0.302
Pancreatic subtypes	96	ND ID:ND	*0.070*^*a*^	*0.058*^*a*^
***Pancreatic adenocarcinoma***	***146***	***TCGA ID:PAAD***	***0.016***^***b***^	0.928
Pancreatic ductal adenocarcinoma	102	GEO ID:GSE21501	0.615	0.598
Skin cutaneous melanoma	468	TCGA ID:SKCM	0.192	0.493
Stomach adenocarcinoma	415	TCGA ID:STAD	0.218	0.738
Thymoma	120	TCGA ID:THYM	0.952	0.113

Bold italic indicates the p value below 0.05

italic indicates the p value between 0.05 and 0.1

*ND* no data

^a^High is worse

^b^High is better

### Analysis of the relation between PFKFB3 and PFKFB4 expression in neuroblastoma patients

**Fig. 1:**
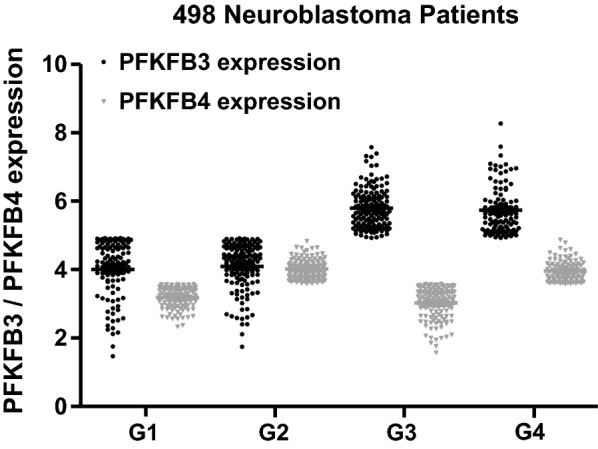
Analysis of PFKFB3 and PFKFB4 mRNA expression in neuroblastoma patients using a publicly available data set GSE62564. 498 neuroblastoma patients were classified in accordance to their PFKFB3 and PFKFB4 mRNA expression. The patients were divided into four expression groups (G1—low PFKFB3/low PFKFB4, G2—low-PFKFB3/highPFKFB4, G3—high PFKFB3/low PFKFB4, G4—high PFKFB3/high PFKFB4) based on the median

**Fig. 2:**
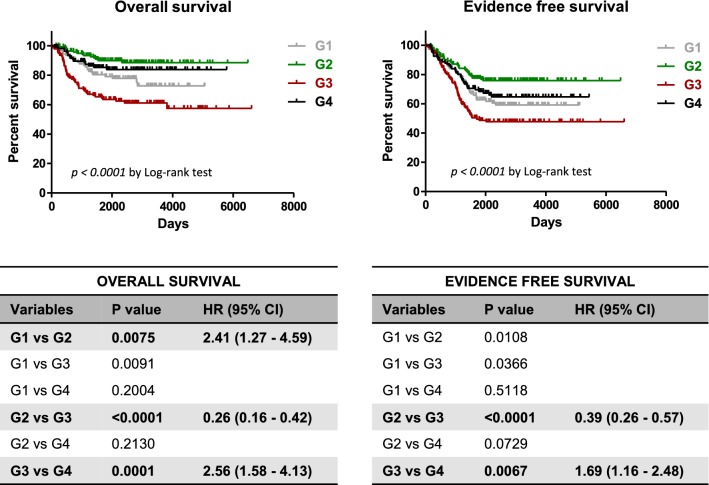
PFKFB3/PFKFB4 expression profile influences the prognosis of neuroblastoma patients. Upper panel: The publicly available data set GSE62564 was used to analyze the overall survival and evidence free survival in four expression groups G1, G2, G3, G4. Kaplan–Meier curves are presents for overall survival and evidence free survival. Lower panel: Survival curves comparison using the log-rank test with Bonferroni correction (p value below 0.0083 was considered significant). Univariate hazard ratios with confidence intervals were obtained by Cox regression analysis

Without doubt, the most interesting data was obtained through neuroblastoma data sets. Surprisingly, in two independent cohorts we observed that high expression of PFKFB3 negatively influences the overall survival, whereas in five independent data sets patients with high expression of PFKFB4 had significantly better OS (Table 1). In order to determine the influence of PFKFB3/4 co-expression on survival rate, the gene expression data were downloaded (GSE62564) and neuroblastoma patients were divided on four groups based on the median PFKFB3/PFKFB4 expression (Fig. 1), as described in Materials and Methods. As determined in Fig. 2, our analysis clearly indicates that patients with high PFKFB4 expression (G2 and G4 groups) have a significantly better overall survival, which might suggest that regardless of the PFKFB3 level of expression (high in G4 group and low in G2), PFKFB4 has a positive impact on the patients survival rate. The worst survival prognosis could be observed for the patients with the dominant PFKFB3 expression (G3 group), which clearly indicates that low expression of PFKFB4, and high expression of PFKFB3 at the same time has a negative impact on the survival of neuroblastoma patients. Importantly, the same correlation was observed for evidence free survival. This suggests that if the high expression of the PFKFB3 isoenzyme is not balanced by the expression of PFKFB4, then the patients show much worse survival rate. Subgroup analysis was further extended, and univariate Cox proportional hazards regression was performed. As determined in Fig. 2 lower panel, there is a better overall survival and evidence free survival rate in the group of patients with dominant PFKFB4 (G2 group) in comparison to the group of patients with dominant PFKFB3 (G3 group), with hazard rates of 0.26 and 0.39 respectively. What is more, group G3 presented worse overall survival and evidence free survival in comparison to the G4 group (high PFKFB3/high PFKFB4), with hazard ratios of 2.56 and 1.69 respectively, which indicates that negative impact of PFKFB3 expression can be offset by the expression of PFKFB4.

## Discussion

Currently, several literature data indicates PFK-II as a potential marker of cancer prognosis [19–25]. Nevertheless, up to this day, most research on PFK-II refers to one of the two cancer specific isoenzymes PFKFB3 or PFKFB4 [8–12, 19, 20, 22–25], undoubtedly neglecting the importance of co-expression of individual isoenzymes. Our analysis of publicly available data sets unquestionably indicates that in several tumors, high PFKFB3 and/or PFKFB4 expression correlates with poor survivability. Importantly, our analysis confirmed the previously published negative impact of PFKFB4 on the prognosis of glioma patients [23] and negative impact of PFKFB3 on the prognosis of hepatocellular cancer [24]. Interestingly, recently published data also revealed the negative impact of PFKFB4 on breast and bladder cancer patients but it was not noticable in the data sets we have analyzed. Nevertheless, Li et al. [22] and Ling et al. [20] analyzed the expression of PFKFB3 and PFKFB4 at the protein level in lung and breast cancer, which definitely may explain the discrepancy between the outcomes. Moreover, it may suggest that the same analysis, as we have done so far, could be performed at the protein level revealing more tumors for which expression of PFKFB3/PFKFB4 constitutes a potent prognostic marker. Undoubtedly, attention should be paid to the fact that in the case of neuroblastoma solely high expression of isoenzyme PFKFB3 correlates with poor prognosis whereas solely high expression of PFKFB4 is a positive prognostic factor for these patients. According to our current knowledge, there is no single study analyzing the influence of PFK-II on neuroblastoma patients’ prognosis, whereas our analysis clearly indicates that cancer specific isoenzymes may have opposite effects on prognosis in this group of specific cancer patients.

Although both genes code for phosphofructokinase II, according to the literature, kinase activity dominates in the PFKFB3 isoenzyme [26, 27], enhancing the glycolytic breakdown of glucose, whereas PFKFB4 seems to have higher FBPase-2 activity [5, 28], stimulating the flow of glucose toward the pentose phosphate pathway, providing the source of NADPH, crucial as a reducing factor for lipid biosynthesis and ROS-detoxifying enzymes. Altogether, we speculate, that the group with high PFKFB3 expression and low PFKFB4 may be characterized by higher glycolysis rates in relation to other groups whereas the PFKFB4 solely high group will have the lower glycolytic activity in comparison to other patients. If PFKFB3 dependent glycolytic cell activity in neuroblastoma indeed has significant impact on neuroblastoma cell proliferation [29], as determined by Almeida et al., one would expect to have opposite effects of PFKFB3 and PFKFB4 expression on patient survival as observed in our study. In addition, the analysis of the impact of isoenzymes on patients’ survival rates should also take into account that both, PFKFB3 and PFKFB4 are vastly involved in other important biological processes in a non-glycolysis-dependent manner [13–16, 30–32], which could also explain their opposite effect on neuroblastoma patients’ prognosing. For instance, it has been shown that PFKFB3 can be localized in the nucleus, which results in proliferation enhancement without increasing of the glycolytic rate. As reported by Yalcin et al. both, kinase activity of PFKFB3 and nuclear localization are needed for its effect on cancer cell proliferation [32]. As for PFKFB4, it has been reported that this enzyme is somewhat involved, be it directly or indirectly, in phosphorylation of the CBP-interacting domain of oncogenic steroid receptor coactivator-3 (SRC-3), enhancing its transcriptional activity, which results in higher expression of its target genes; transketolase, adenosine monophosphate deaminase-1 (AMPD1) and xanthine dehydrogenase (XDH), involved in the metabolism of nucleotides [13]. Regardless of the molecular mechanism of action of PFKFB3/4 isoenzymes our data clearly indicates that in analyzing the effect of PFK-II on the prognosis of cancer patients, the expression of both PFKFB3/4 isozymes should be considered. This is particularly important in the case of potential use of PFK-II inhibitors, because, as our analysis has undoubtedly shown, the effectiveness of inhibition may be dependent on the suitable inhibitor application for a specific isoenzyme overexpressed in specific cancer cells. What is more, our analysis indicates worryingly, that application of PFKFB4 inhibitors for neuroblastoma patients characterized by overexpression of both isoenzymes could even have a negative impact on the patient prognosis. Consequently, the results of our analysis provide important insight for future clinical oncology by presenting the importance of metabolic enzymes as a likely destination for modern targeted anti-tumor therapy.

## Data Availability

The datasets used and/or analysed during the current study are available in the R2 database repository, https://hgserver1.amc.nl/cgi-bin/r2/main.cgi, date of access 12.02.2019. Gene expression and survival data of 498 neuroblastoma patients were downloaded from the NCBI Gene Expression Omnibus (GEO), a public repository of microarray data, dataset ID: GSE62564.

## Abbreviations

AMPD1: adenosine monophosphate deaminase 1
ATP: adenosine triphosphate
GEO: gene expression Omnibus
FBPase-2: fructose-2,6-biphosphatase
NADPH: dihydronicotinamide-adenine dinucleotide phosphate
OS: overall survival
PFK-I: phosphofructokinase I
PFK-II: phosphofructokinase II
PFKFB: 6-phosphofructo-2-kinase/fructose-2,6-biphosphatase
PFKFB3: 6-phosphofructo-2-kinase/fructose-2,6-biphosphatase 3
PFKFB4: 6-phosphofructo-2-kinase/fructose-2,6-biphosphatase 4
ROS: reactive oxygen species
SRC-3: steroid receptor coactivator-3
XDH: xanthine dehydrogenase
